# Medial injuries of the clavicle: more prevalent than expected? A big data analysis of incidence, age, and gender distribution based on nationwide routine data

**DOI:** 10.1007/s00068-019-01293-0

**Published:** 2020-01-20

**Authors:** Mustafa Sinan Bakir, Jan Unterkofler, Lyubomir Haralambiev, Simon Kim, Roman Carbon, Axel Ekkernkamp, Stefan Schulz-Drost

**Affiliations:** 1 Department of Trauma and Reconstructive Surgery and Rehabilitative Medicine, Medical University Greifswald, Ferdinand-Sauerbruch-Straße, 17471 Greifswald, Germany; 2 Department of Trauma Surgery and Orthopedics, BG Hospital Unfallkrankenhaus Berlin gGmbH, Warener Straße 7, 12683 Berlin, Germany; 3 Department of Vascular Surgery, University Hospital RWTH Aachen, Pauwelsstraße 30, 52074 Aachen, Germany; 4 Department of Pediatric Surgery, University Hospital Erlangen, Krankenhausstraße 12, 91054 Erlangen, Germany; 5 Department of Trauma and Orthopedic Surgery, University Hospital Erlangen, Krankenhausstraße 12, 91054 Erlangen, Germany; 6 Department of Trauma, Orthopedic and Hand Surgery, Helios Hospital Schwerin, Wismarsche Straße 393-397, 19049 Schwerin, Germany

**Keywords:** Shoulder-girdle, Sternoclavicular joint dislocation, Medial clavicle fracture, Incidence, Gender, Age distribution

## Abstract

**Purpose:**

Although shoulder-girdle injuries occur frequently, injuries of the medial part remain widely unexplored. This study overviews these rare injuries with a focus on incidence, age, and sex distribution in Germany.

**Methods:**

The data are based on diagnoses according to ICD-10 in all German hospitals provided by the German Federal Statistical Office. ICD-10 codes S42.01 (medial clavicle fracture, MCF) and S43.2 (sternoclavicular joint dislocation, SCJD) were evaluated in detail between 2012 and 2014.

**Results:**

We identified 14,264 cases with medial clavicle injuries (MCIs). MCFs occurred more often (11.6% of all clavicle-related shoulder-girdle injuries vs. 0.6% for SCJD). Mean ages of MCI were significantly different between males (43.7 years) and females (57.1 years) (*p* < 0.01). Age demonstrated a bimodal distribution with peaks at 20 and 50 years, which were predominantly associated with males. Females showed more injuries at age beyond 70 years. This applies to both SCJD and MCF. The incidence rate of these shoulder-girdle injuries was 47.0 per 100,000 person-years, for MCIs overall 5.9 (4.1 for men, 1.8 for women). This indicates disparity with a significant predominance of male patients over females as for all shoulder-girdle injuries (*p* < 0.01). Among children (< 16 years old), the incidence rate showed no significant difference in gender ratio.

**Conclusion:**

MCIs appear more frequently than estimated so far and are distinguished from other clavicle fractures in that they occur more at higher age and peaking around 50 years. Further work on possible prevention strategies should focus on the most frequently affected groups of men around 20 and 50 years old.

## Introduction

Among trauma patients, clavicle fractures appear as one of the frequent injuries and represent about 2.5–10.0% of all fractures in general [1–3]. However, medial clavicle fractures (MCFs) and sternoclavicular joint dislocations (SCJDs) are generally considered rare injuries [2–8]. Only about 5% of clavicle fractures occur in the medial clavicle, whereas the incidence of mid-shaft fractures is over 65–80% [7–9]. The incidence of medial clavicle injuries (MCIs) is 3% of all shoulder-girdle injuries, including injuries of the clavicle and the sternoclavicular and acromioclavicular joints. Solitary clavicle fractures represent about 44% of shoulder-girdle injuries, including injuries of the proximal humerus and rotator cuff [2, 6].

The medial part of the clavicle plays a special role as a link between the upper extremities/shoulder-girdle and the thorax/trunk [10]. Such injuries are predominantly caused by high impact [2, 6, 11, 12]. Despite the low incidence, these injuries are important because of their severity and the potentially serious acute and chronic complications, such as vascular, nerve, or tracheal lesions and posttraumatic arthrosis [6, 11].

Younger patients are disproportionately affected by clavicular fractures in general [2, 13]. In addition, men, especially young adults, are the largest single cohort in this regard [2, 13]. However, scientific reports on the age distribution, gender distribution, and incidence of MCI are rare [1]. Published case reports regarding MCI show a heterogeneous pattern of age structure, where patients are mainly in their 20 s or 45–59 years old [14, 15]. The primary objective of this study is to show the importance of SCJD and MCF in clavicle involving shoulder-girdle injuries relative to their incidence. Our secondary goal is to present the age distribution of MCI and to answer the question of whether men are more frequently affected than women and whether there is an age difference concerning transport vehicle accidents.

## Methods

The cohort comprises all released inpatients (including the deceased) from all German hospitals according to diagnosis-related groups (DRGs) in the scope of § 1 of the German Hospital Finance Law (KHEntgG) [16]. The routine data were provided by the German Federal Statistical Office and based on the 10th revision of the International Statistical Classification of Diseases and Related Health Problems (ICD-10 codes) [17]. We analyzed ICD-10 codes S42.01, S42.02, and S42.03 (clavicle fracture of the medial, midshaft, and lateral third, respectively), in addition to S43.1 and S43.2 (acromioclavicular and sternoclavicular joint dislocations, respectively) from 2012 to 2014. We retrospectively analyzed the incidence, age, and sex distribution with a focus on the medial clavicle injuries S43.2 and S42.01. The number of injuries according to transport vehicle accidents as the responsible accident cause was additionally identified via ICD-10 code V99. These codes include several transport vehicles such as motor vehicle accidents (car, truck, train, motorbike), pedestrians hit by motor vehicle, bicycle and horse accidents, and furthermore on water, on land and in the air. The study was approved by the local ethics committee (BB 007/19), has been performed in accordance with the ethical standards laid down in the 1964 Declaration of Helsinki, and was registered with the German Clinical Trials Register (DRKS; DRKS00017018).

The provided ICD-10-related age data is divided by the German Federal Statistical Office into 5-year age ranges except for ages less than 1 year and greater than 95 years. In contrast, the data of the respective population size are divided into varying age intervals differing from the referred 5-year age ranges. Therefore, these age intervals have been adjusted and harmonized accordingly. The average age was calculated based on the mean of the 5-year age range since there was no possibility of tracing the exact age because of missing individual data. An analysis was done for both men and women and for a gender-neutral cohort.

For incidence rate calculations, the injured patients were compared to the population data of the German Federal Statistical Office, and the results were reported as injuries per 100,000 people per year (person-years). To calculate the incidence rate ratio, the lower value of the incidence rate was used as a reference. A statistical analysis was performed using SPSS software (IBM SPSS Statistics for Windows, Version 22.0. Armonk, NY: IBM Corp.). Associations were tested by Pearson’s chi-squared test. Fisher’s exact test with an alpha-level of 0.05 was used in the case of expected cell values less than *n* = 5. The mean values were compared for unpaired samples using the Student’s *t* test. Due to the explorative nature of the analysis, no alpha adjustment for multiple testing was conducted.

## Results

**Fig. 1:**
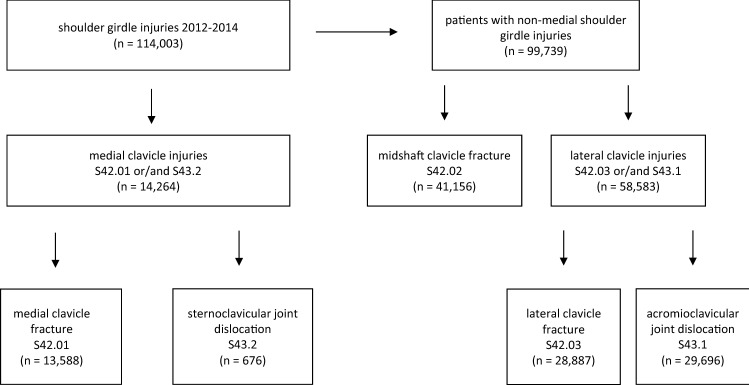
Prism of distribution of shoulder-girdle injuries involving the clavicle in 2012–2014 according to the anatomical location. *n* number of patients

We reviewed a total of 114,003 patients who were diagnosed with a clavicle injury (Table 1). Encompassing all clavicle injuries, 12.5% of these were coded as MCI for a total of *n* = 14,264 patients (Fig. 1). The group of MCFs (S42.01) and SCJDs (S43.2) was used for further investigations. The mean age was slightly different with 50.3 (± 23.3) years for SCJDs and 47.7 (± 22.8) years for MCFs (MCI in total: 47.8 (± 22.8) years). Men with MCI were significantly younger than women with an average age of 43.7 (± 20.3) years for males and 57.1 (± 25.3) years for females (*p* < 0.01).

**Table 1:** Distribution of clavicle-related shoulder-girdle injuries presented as absolute and relative numbers

	Medial to lateral					
ICD 10 diagnosis	S43.2	S42.01	S42.02	S42.03	S43.1	Total
Total (*n*)	676	13,588	41,156	28,887	29,696	114,003
Total (%)	0.6	11.9	36.1	25.3	26.1	100.0
IR total	0.28	5.6	17.0	11.9	12.2	47.0
Male (*n*)	454	9,427	30,631	17,710	25,735	83,957
Male (%)	0.5	11.2	36.5	21.1	30.7	100.0
IR male	0.38	7.9	25.8	14.9	21.7	70.7
Female (*n*)	222	4,161	10,524	11,176	3,958	30,041
Female (%)	0.7	13.9	35.0	37.2	13.2	100.0
IR female	0.18	3.4	8.5	9.0	3.2	24.3


The total amount and the distributions of males and females are demonstrated with an illustration of the injury’s anatomical location. Male patients are significantly more frequently represented in all diagnosis types than females (*p* < 0.01)

*n* number of patients, *%* percentage of all shoulder-girdle injuries relating to the clavicle, *S43.2* sternoclavicular joint dislocations, *S42.01* medial clavicle fracture, *S42.02* midshaft clavicle fracture, *S42.03* lateral clavicle fracture, *S43.1* acromioclavicular joint dislocation, *IR* incidence rate reported as injuries per 100,000 person-years

**Fig. 2:**
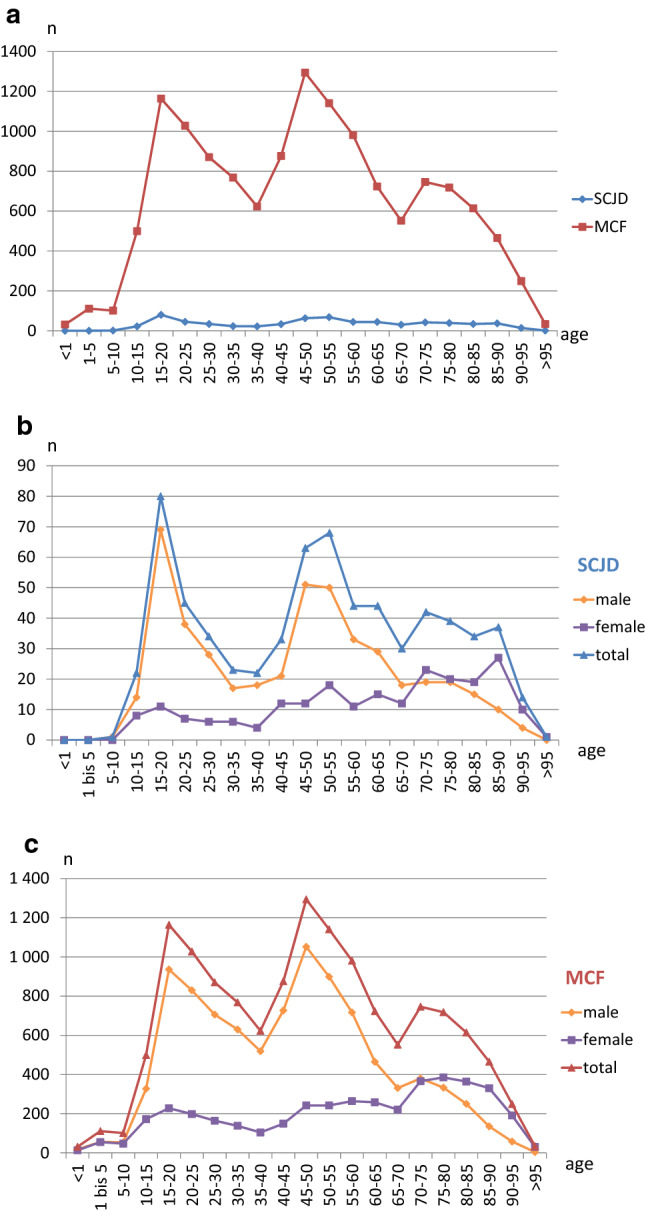
Age distribution of medial clavicle injuries (MCI) divided into sternoclavicular joint dislocations (SCJD) and medial clavicle fractures (MCF). Both MCIs are presented together in a diagram as an overview (**a**), for sternoclavicular joint dislocations only (**b**), and for medial clavicle fractures only (**c**) each in detail. The data are shown separated by gender and for men and women summed up. The age data is divided into ranges of 5-year periods. *n* number of patients

The age distribution demonstrated a bimodal distribution with peaks at 15–20 years and 45–50 years (Fig. 2a). Males were more often associated with both peaks, while females made up a higher proportion of injuries among patients older than 70 years (Fig. 2b,c). This applies to both SCJD (Fig. 2b) and MCF (Fig. 2c). Men were affected much more frequently by these injuries than women.

The incidence rate was 47.0 per 100,000 person-years for all analyzed shoulder-girdle injuries and 34.5 per 100,000 person-years for the subgroup of clavicle fractures (Table 1). The incidence rate of MCI was 5.9 per 100,000 person-years overall, 8.3 per 100,000 person-years for men, and 3.5 per 100,000 person-years for women. These incidence rates were significantly different and showed an incidence rate ratio (IRR) of 2.35 (*p* < 0.01). The IRR shows further gender disparities comprising a significant over-representation of male patients with an IRR of 2.32 (*p* < 0.01) for all clavicle fractures and an IRR of 2.91 (*p* < 0.01) for all shoulder-girdle injuries. IRRs between 1.65 and 6.77 (Table 1) demonstrate that male patients were significantly more frequently affected in all diagnoses than female patients (*p* < 0.01).

The incidence rates of the age cohorts are shown in Table 2. The highest incidence rate was found for both SCJD and MCF for males and for both sexes in the age group of 15–21 years. Among female patients, the highest incidence rates for both MCIs were in the age range of 60–65 years. In contrast, the IRR of the gender distribution was different for the respective MCIs. In the case of SCJD, the IRR was lowest at the ages of 10 to under 15 years (IRR = 1.66), and the highest was at the ages of 15–21 years (IRR = 5.94). However, MCF showed the lowest IRR in children under 10 years old (1.06) and the highest IRR in the age group of 30–50 years (4.47). Among children (< 16 years old), the incidence rate showed no significant difference in gender ratio in contrast to adults.

**Table 2:** Incidence rates and age distribution of medial clavicle injuries for sternoclavicular joint dislocation (S43.2) and medial clavicle fracture (S42.01) for males and females

Age in years		< 10	10–15	15–21	21–30	30–50	50–60	60–65	> 65
S43.2	Total	0.00	0.20	0.54	0.17	0.03	0.18	0.29	0.06
	Male	0.01	0.24	0.91	0.28	0.05	0.27	0.39	0.08
	Female	0.00	0.15	0.15	0.05	0.02	0.10	0.19	0.04
S42.01	Total	1.17	4.43	7.92	3.87	1.16	3.07	4.74	1.09
	Male	1.20	5.66	12.40	6.10	1.89	4.83	6.27	1.52
	Female	1.14	3.14	3.19	1.52	0.42	1.30	3.30	0.77

The incidence rate is reported as injuries per 100,000 person-years. The higher end of the age interval is exclusive (e.g., “10–15”: ages of 10 years to under 15 years)

**Fig. 3:**
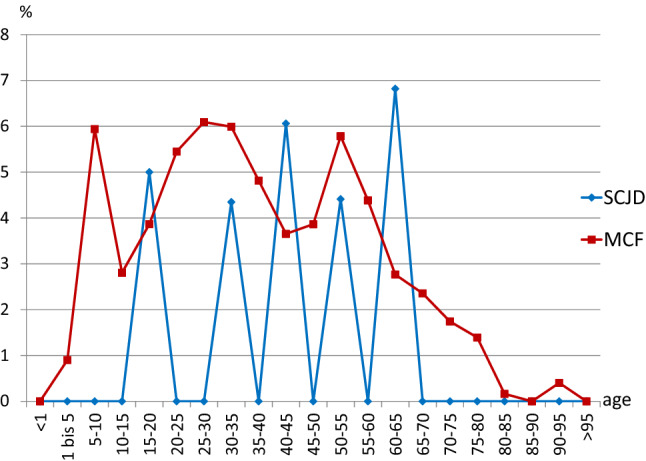
Age distribution of medial clavicle injuries (MCI) caused by transport vehicle accident. Data are divided into sternoclavicular joint dislocations (SCJD) and medial clavicle fractures (MCF). The age data are divided into ranges of 5-year periods. *%* percentage of patients additionally coded with transport vehicle accident

Analysis of trauma mechanism showed similar results but statistically significant differences in transport vehicle accidents between MCIs (3.6%) and lateral clavicle injuries (3.1%) (*p* = 0.001). Focussing MCIs, we found a statistically significant difference between the two entities with 1.9% for SCJD and 3.7% for MCF. Divided into age groups the peaks in transport vehicle accident-related MCFs were in older childhood, between 20 and 35 years and at age from 50 to 55 years (Fig. 3). For SCJD, the coded association with transport vehicle accident showed a heterogeneous pattern between 15 and 65 years.

## Discussion

Of all clavicle injuries, 0.6% were SCJDs, and 11.9% were MCFs, which differ slightly from past data. The proportion of SCJD was found to be higher, and the incidence of MCFs was found to be lower [2, 5, 6]. MCI in total seems to be more prevalent than expected so far with a proportion twice as large as previously described [2–9]. The average age was only slightly lower at 47.8 years than in previous clinical research on MCI in Germany (54.9 years) but was the same mean age as in a Swedish study concerning clavicle fractures [9, 12].

The age pattern was also similar with less deviation of a peak in old age over 80 years in a previous clinical study, which included only a small number of cases (*n* = 19) [12]. The present data are based on a larger sample, so the results might be considered more representative. The expected predominance of men in the incidence of MCI was confirmed [12]. The significantly higher incidence among men also applies to all types of shoulder-girdle injuries investigated. The significant difference in mean age for MCI among men was about 13 years lower than among women, which is similar to previous clavicle fracture data from Belgium [18].

There is limited published data on the epidemiology of medial shoulder-girdle injuries, but the incidence rates of fractures of the clavicle can be compared with previous studies [13]. Our incidence of all clavicle fractures in Germany (34.5 per 100,000 person-years) is about 45% higher than the incidence rate in the US from 2002 to 2006 (24.4 per 100,000 person-years). However, the gender ratio is only slightly different [13]. The sex ratio for MCI as a subgroup is also similar between our study and clavicle fracture data from the US and Sweden [9, 13]. Compared with European data from Belgium in 2015 (70.6 per 100,000 person-years), the amount in our study for clavicle fractures only was 50% of the Belgian incidence rate [19]. Some of the differences could be due to the different time intervals analyzed between studies since Herteleer et al. demonstrated an increasing incidence rate from the recent past to the present [13, 19].

The age distribution differs from previous results from Italy and the USA concerning only or predominantly midshaft clavicle fractures [2, 13]. The peaks in our study were at 20 and 50 years, which are mainly attributed to male patients, whereas MCI often occurs in females at 70–80 years. In the Italian and US epidemiologic analyses, however, the highest incidence was seen in the first 2 decades for both men and women [2, 13]. On the other hand, our MCI data concerning age distribution shows an analogical bimodal pattern that is consistent with peaks in Belgian data [18]. The higher incidence in male patients was confirmed in comparisons with previous data [1, 2, 9, 13, 18, 20]. High-impact trauma is associated with male risk-taking behavior, and this kind of trauma mechanism is a common cause for shoulder-girdle injuries, which could be a reason for our age and sex-pattern findings [12, 13, 21, 22].

The additional peak around the age of 50 years and the relatively high incidence in old age distinguishes MCI from other clavicle fractures. Thus, the results confirm the speculation by Postacchini et al. that these types of injuries occur quite often at advanced ages over 65 years [2]. This finding coincides with the distribution of MCI (and partly for clavicle fractures in general) from Swedish and Belgian studies [1, 9, 18–20]. An alternative potential factor for the present peak in both men and women around the age of 50 years may be a demographic peculiarity. This refers to the generation of so-called "baby boomers," which had a significantly higher birth rate in Germany as well [23]. This fact generally has to be considered in epidemiological issues in Germany since a higher incidence in all types of injury has to be expected due to the larger generation size. However, as this peak is also found in other European studies, this bias can be considered to be of secondary relevance [1]. Furthermore, we set the respective cases of injury in relation to the size of the age cohorts to decrease bias by preventing distortion due to a differently sized and differently distributed population structure.

In general, the use of incidence rates as an alternative to the absolute numbers allows for further comparisons and prevents distortion due to a differently distributed population structure. In our study, the incidence rates displayed a comparable picture to the absolute figures. The highest incidence rates of MCI were in the age group of 15–21 years for men and for both sexes together, while the highest incidence rate in female patients occurred for ages of 60–65 years. Our analysis showed the lowest gender differences in MCI before adulthood. With increasing age, the difference emerged, which is quite plausible with regard to the gender roles, which are not present in early childhood [24].

The turning point at which the ratio tilts and the number of cases of MCI among women exceeds that of men is around the age of 70 years. This result is also consistent with the age-stratified gender distribution of overall clavicle fractures [9, 13, 18]. Despite the higher number of cases, even in old age, the incidence in female patients is always lower than in male patients due to the disproportionately larger proportion of women at the age of 60 years and beyond in the total population according to the German Federal Statistical Office data. Since they have a longer life expectancy, the cases of MCI are distributed among a disproportionately larger cohort [25].

The proportion of MCIs caused by transport vehicle accidents may be related to risk-taking behavior, too, which is more likely to be associated with males [12, 13, 21, 22]. However, a differentiation of the different types of transport vehicle accidents is not possible, since these are only shown cumulatively in the ICD-10 code [17]. Therefore, no reliable, meaningful conclusion can be derived from this part of the analysis. The confession of a limited validity can also be underlined by the low proportion of injuries caused by transport vehicle accidents. This differs distinctly from previous work in which the proportion of this trauma mechanism is more than 60% [12, 26, 27]. Presumably, one reason for this underrepresentation is that the V99 coding for motor vehicle accident is merely an additional coding and not a necessity, but only a supplement [17]. Thus, we assume a rather low passion of the coders for a highly detailed coding. Our hypothesis that MCIs are more frequently caused by a high-impact trauma mechanism than lateral clavicle injuries was statistically confirmed and corroborated. This finding is consistent with those of previous studies [12, 13, 21, 22]. Regarding an explicit conclusion on trauma mechanism, we can only assume that sports and motor vehicle accidents are main reasons for the male MCI peaks and the female MCI peak is attributed to falls in older women with osteopenia.

A limiting and possibly causative factor for the difference in clavicle fracture incidence between Germany and Belgium is that our statistical analysis only includes inpatients. The underreporting of outpatient cases might be quite common and probable, especially in uncomplicated and conservatively treated injuries [19]. This fact could influence the age distribution by leading to an overestimation of the higher ages since patients treated in an outpatient setting are also usually the more healthy ones and therefore often younger. An over-reporting of complex injuries, which are more often the medial injuries compared to the further clavicle injuries, could also be caused by this selection bias. An incorrect and biased distribution of the clavicle-related shoulder-girdle injuries might be a potential consequence. On the other hand, over-reporting might be possible due to cases of one diagnosis involving several inpatient admissions, as in cases of infection or the removal of osteosynthesis material, for example. This could lead to duplicate counting of the same patient because the original main diagnosis is always added in these cases. For further studies, the role and amount of osteosynthesis material removals and therapeutic procedures, in general, should be analyzed.

Previous work with a large cohort concerning clavicle fractures was partly limited by a lack of differentiation between the locations of the fractures [13]. Our large demographic sample size should provide power to the incidence analysis, but the missing opportunity for double-checking between coding and the correct radiologic location could be a potential bias [12]. The data are only as precise as the accuracy of the person entering it. Data quality is reduced, for example, by the use of superordinate and simpler coding such as S42.00 for a clavicle fracture in general without further description [17]. In addition, there is some inaccuracy of the diagnosis since this coding merely divides into a medial, middle or lateral third of the clavicle location. A more exact classification, e.g., according to Robinson [5] or Bakir et al. [12], is not possible due to the lack of traceability of the individual cases. These biases are a weakness of all work analyzing routine data and could be a factor in this study as well.

Nonetheless, since our study covers the entire German population, an all-encompassing picture emerges in comparison to previous analyses concerning the epidemiology of MCI, which originated from only a single city [1]. Therefore, we can draw conclusions about the total population of a modern industrial nation with both urban and rural areas. Possible prevention strategies should particularly aim at these commonly affected target groups, which are predominantly males.

## Conclusion

The high number of cases in this study implies that a reliable statement about the age and gender distribution of MCI can be made. SCJDs are an unusual injury, and the incidence of this injury compared with all clavicle-related shoulder-girdle injuries seems to be extremely rare and even less frequent than assumed [4, 6, 11, 15]. In contrast, MCF occurs more frequently than estimated so far. MCI shows a clear predominance in men in terms of the incidence of both SCJD and MCF. The age distribution shows a gender-dependent pattern with peaks in men around 20 and 50 years old and in women between 70 and 80 years old, when they surpass the males in this age group. With regard to all clavicle fractures in total, the incidence would be located in an international ranking comparison in the midfield. A decreasing incidence of injuries via focused prevention might be just as possible as a reduction of negative long-term consequences of missed MCI through a focused examination of the target groups. Despite the opportunity to achieve good results by analyzing large cohorts, as demonstrated in our work, the emerging studies working with big data should always keep in mind the limitations of (inpatient) selection bias, data quality and input/coding quality.
